# Growing a garden of neurons

**DOI:** 10.3389/fninf.2013.00017

**Published:** 2013-08-26

**Authors:** Rebekah C. Evans, Sridevi Polavaram

**Affiliations:** Center for Neural Informatics, Krasnow Institute for Advanced Study, George Mason UniversityFairfax, VA, USA

**Keywords:** morphology, dendrites, growth algorithm, computational model, neural networks

Computational models of biologically realistic neuronal networks have advanced neuroscience in the past 20 years. With an ultimate goal of simulating a whole brain, these networks must become larger and more complex. However, a sheer massive number of neurons do not make a brain. Neurons are all different, with different kinetics, neurotransmitters, and importantly different morphologies. A network can be made by connecting copies of the same cell together, but this kind of *homogenous* network can only explain so much. Real neuronal networks are *heterogeneous* and are made up of neurons that follow both intrinsic and extrinsic cues to grow their unique dendritic arbors (Scott and Luo, 2001). In addition to *homogenous* and *heterogeneous* network models, *hybrid* network models have been implemented by creating a small heterogeneous network and replicating it to establish a larger network (Kozloski, 2011). However, modeling studies have shown that homogenous networks act differently than realistic heterogeneous ones (Mäki-Marttunen et al., 2011). Because computational neuronal networks need to grow larger to simulate complete brain regions, and because heterogeneity in a network is critical to modeling a realistic brain, algorithms for digitally generating neural morphologies are a necessary step toward this goal.

A new paper by Memelli et al. (2013) joins the field of papers providing algorithms for growing digital neurons. Their algorithm can be used to build a network consisting of millions of neurons each with a unique morphology. The current models, L-Neuron (Ascoli et al., 2001), NeuGen2.0 (Wolf et al., 2013), NetMorph (Koene et al., 2009), and CD3X (Zubler and Douglas, 2009) have made great strides in advancing the process of generating digital neurons. These models are all publicly available, and can be used to generate large networks of neurons. Recently L-Neuron was used to generate a 0.5 million cell model of the dentate gyrus (Schneider et al., 2012). Each algorithm has its own specific advantages. NetMorph has a synapse-generating algorithm, NeuGen2.0 is modular and adaptable to new data, and CD3X can isolate intrinsic and extrinsic factors of neuron development by growing the same neurons in different model environments. In combination with the parallelization of simulation software [such as NEURON (Migliore et al., 2006)], these neuron generators are laying the groundwork for enabling massive biologically realistic simulations.

Memelli et al. (2013) do not attempt to model the molecular mechanisms of dendritic growth, but instead work to make a concise, computationally efficient model that can capture the structure and variability of realistic morphologies. Their work adds two elements to this field. First, it simplifies the neural growth algorithm to contain a combination of three biologically inspired intrinsic parameters: soma-oriented tropism, dendritic self-avoidance, and membrane stiffness. The three parameters of their growth algorithm are all intrinsic to the cell itself and do not take into account any extrinsic signals that could come from other neurons or physical constraints. Each of these parameters has been previously described, but Memelli et al. are the first to combine them in one simple model. Second, their algorithm is written to be fast and massively parallel, creating the possibility for generating billions of neurons on the IBM Bluegene computer. Their algorithm can generate a neuron in less than two seconds, and when run on parallel cores is capable of generating enough neurons to simulate an entire brain region. Together, these elements fit the need to have morphological diversity within a network as well as the need to have extremely large networks.

Each of the current morphology simulators has their particular strengths. The ideal situation would be for Memelli's new algorithm to be incorporated into one of the existing ready-to-use packages. For example, the application of this algorithm within the external constraints of CX3D could help isolate the extrinsic and intrinsic aspects of dendritic arborization. When used together these simulators can help create massive-scale heterogeneous networks for computational modelers and can help investigate how dendrites actually grow.
